# Dermoscopic clues in the skin lesions of secondary syphilis

**DOI:** 10.1002/ccr3.1999

**Published:** 2019-01-25

**Authors:** Mahesh Mathur, Prakash Acharya, Alina Karki, Jyoti Shah, Nisha KC

**Affiliations:** ^1^ Department of Dermatology College of Medical Sciences Bharatpur Nepal

**Keywords:** Biett’s sign, dermatoscopy, dermoscopy, infectious disease, syphilis

## Abstract

Secondary syphilis may have a varied clinical presentation and might pose a diagnostic difficulty when a typical history is absent. We describe the dermoscopic clues of the skin lesions at different stages of the disease which could culminate to a proper diagnosis.

## 1 INTRODUCTION

Early diagnosis of syphilis is important to prevent serious morbidities associated with the disease. Skin lesions may mimic a wide range of diseases causing diagnostic difficulties, especially when the presentation is atypical. We describe dermoscopic findings in a patient of secondary syphilis who had a history of repeated blood transfusions.

Syphilis remains a global health problem even when the sensitive tests and affordable treatment are available.^1^ This disease has been referred to as the “Great Mimicker” due to its varied clinical presentations which may resemble other infections.^1^ A high level of clinical suspicion of this potentially serious condition is necessary to make a correct diagnosis.^2^ This becomes quite challenging when the skin lesions of syphilis do not follow a typical course, especially when patient presents with the skin lesions suggestive of secondary syphilis without a history of preceding primary genital or extragenital lesions. Dermoscopy may prove to be a useful tool during such circumstances.

To our current knowledge, only a few studies describing the dermoscopic features of secondary syphilis lesions exist in the literature.^3^, ^4^, ^5^ We aim to highlight the use of dermoscope as an auxiliary tool for the identification of skin lesions at different stages of secondary syphilis.

## 2 REPORT OF A CASE

**Figure 1:**
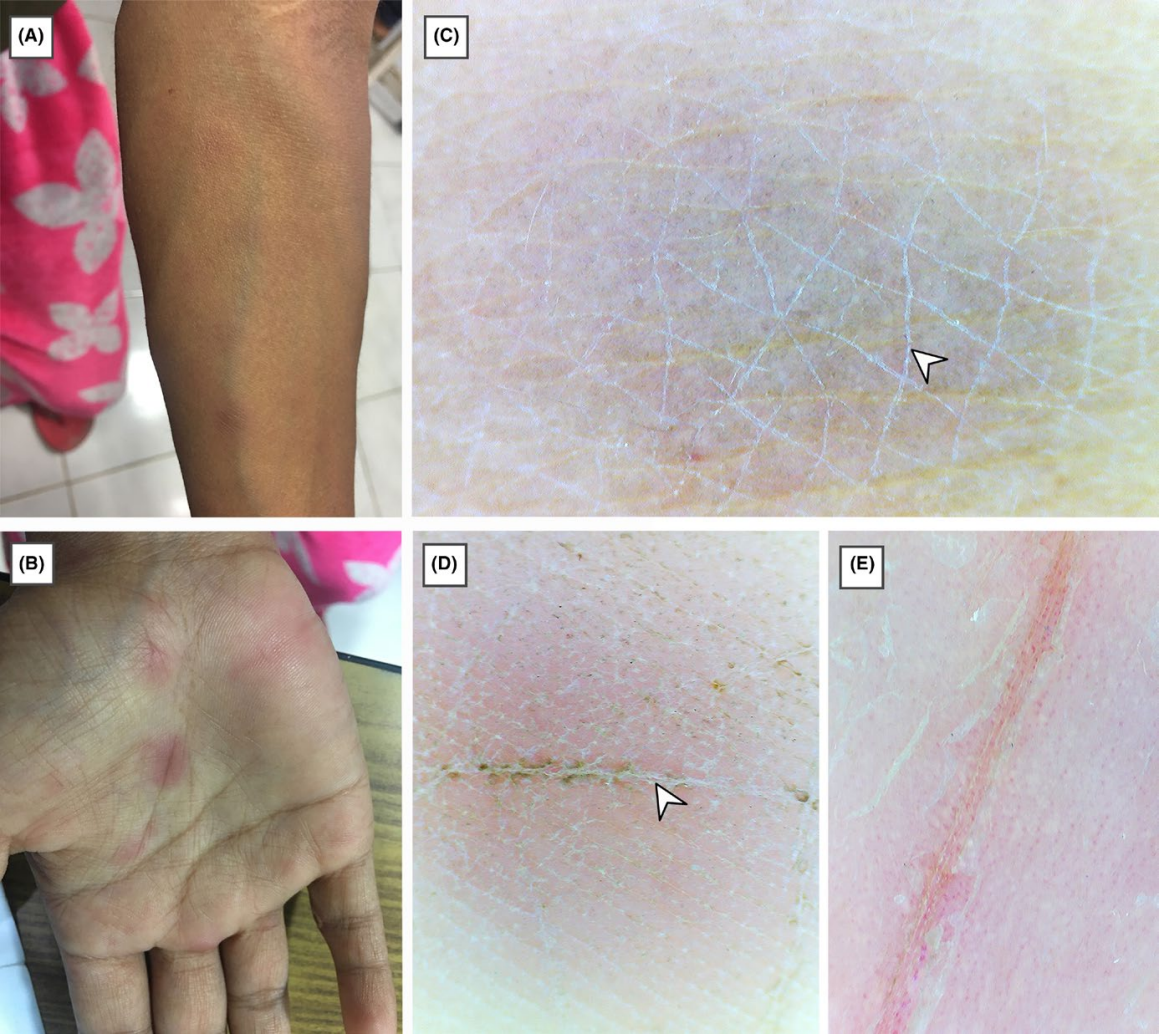
A and B, Clinically, erythematous maculopapular rash can be seen over left forearm and right palm. Dermoscopy (polarized dermoscopy, original magnification × 20) of lesions over forearm, C and palm, D showing scaling within the skin furrows (white arrowheads) and central darker area fading toward the periphery with an ill‐defined border. Orange color of the lesion can be noted in the lesion over palm. E, Dermoscopy (polarized dermoscopy, original magnification ×20) of a psoriatic lesion over palm from a different patient showing larger scales, pinkish color and dotted vessels

An 18‐year‐old unmarried female presented with a 10‐day history of multiple erythematous maculopapular rashes over bilateral upper limbs, trunk, palms, and soles (Figure 1A,B). She was undergoing hemodialysis for chronic renal failure for the last 4 years. During her course of the disease, she had multiple episodes of blood transfusion for anemia. She denied any contact or any skin lesions over the genitalia in the past. Examination of the mucosa showed no abnormalities. Bilateral epitrochlear and inguinal lymph nodes were enlarged, mobile, and nontender.

Polarized light dermoscopy (Firefly Pro, MA, USA) of the lesion on her left forearm and right palm revealed scaling within the skin furrows and central darker area fading toward the periphery with an ill‐defined border (Figure 1C,D). Dermoscopic image of the lesion on the palm was compared to the dermoscopic image obtained from a psoriatic lesion (Figure 1E) over a similar site from a different patient. This was helpful to appreciate the orange color, smaller scales and absent vascular pattern in the syphilitic lesion compared to the pinkish‐red color, larger scales and dotted vessels in the psoriatic lesion.

**Figure 2:**
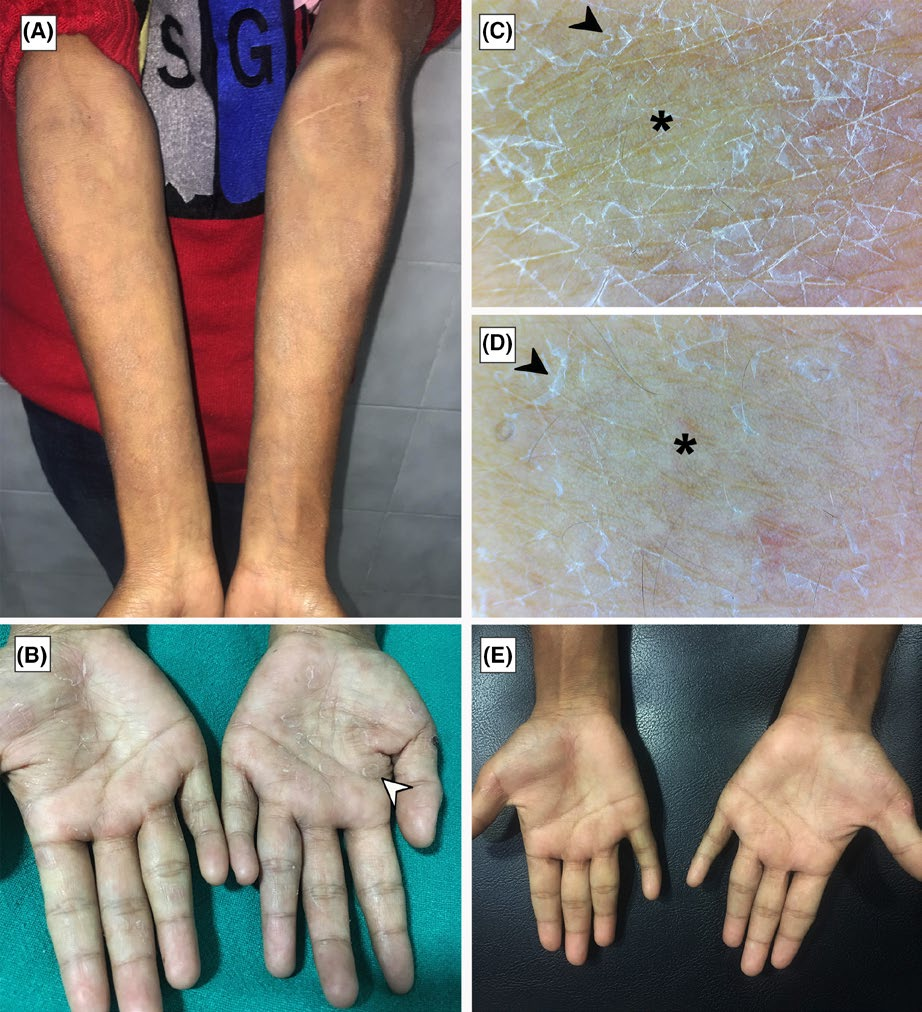
A and B, Clinically, lesions with increased scaling and decreased erythema can be seen over bilateral forearms and palms, respectively, on the 5th day of initial presentation. Peripheral scaling can be clearly noted in the lesions over palms. C and D, Dermoscopy (polarized dermoscopy, original magnification × 20) of lesions over forearm during the visit showing peripheral scaling with a relatively clear central area. E, Clearance of scaling seen on palms on the 14th day of treatment

Serological tests were ordered and the patient presented with the reports five days after the initial visit which showed positive venereal disease research laboratory (VDRL) test (titer of 1:64) and a reactive treponema pallidum hemagglutination assay (TPHA). Based on the clinical features and laboratory findings, the diagnosis of secondary syphilis was made. Examination of lesions over forearms and palms during this visit revealed a reduction in erythema and increased scaling (Figure 2A,B). Dermoscopic evaluation of the lesions over two different sites in the left forearm was done which showed peripheral scaling with a relatively clear central area (Figure 2C,D).

The patient was treated with a single dose of 2.4 million units of benzathine penicillin G given intramuscularly and observed for any complications. Follow‐up on the 14th day of treatment revealed a significant reduction in scaling over most lesions with the complete clearance of lesions in some areas (Figure 2E).

## 3 DISCUSSION

Untreated individuals infected with Treponema pallidum typically follow a course of disease which includes primary, secondary, latent, and tertiary stages.^6^ Early recognition and treatment of syphilis are of prime importance to avoid serious systemic complications.^6^

Secondary syphilis occurs due to the dissemination of the treponeme via blood or lymphatics which occurs 6‐8 weeks after the appearance of primary lesions.^2^ This stage is characterized by the initial appearance of evanescent macular rash followed by symmetric papular eruptions over trunk, extremities, palms, and soles which are generally scaly. Mucosal lesions are common, and condyloma lata may be seen in intertriginous areas.^7^

Our patient was unmarried, had no history of contact and preceding genital or extragenital skin lesions suggestive of primary syphilis, and serology for VDRL was negative before starting hemodialysis. She had a history of repeated blood transfusions for anemia during the course of her systemic disease which led us to the suspicion of transfusion‐associated syphilis infection. However, this may be affected by unreliable history and easily missed primary syphilitic lesions. Only one case of transfusion‐associated syphilis has been reported from the United States in the last 50 years and another case reported from India recently.^8^, ^9^ This markedly lowered incidence is probably due to the testing of donors for syphilis and the inability of syphilis spirochete to survive in refrigerated blood beyond 5 days. Syphilis transmitted due to blood transfusion shows no primary lesions and is termed as Syphilis d’ emblee.^9^ We believe that our patient may be a case of Syphilis d’ emblee.

Studies done previously described the presence of diffuse orangish or yellowish red background with vascularity as the most consistent dermoscopic findings of secondary syphilis lesions.^3^, ^4^, ^5^ Although a diffuse orangish background was observed in the palmar lesion of our patient, a definite vascular pattern could not be appreciated. We found that the dermoscope was useful to visualize the mild scaling usually present in the skin furrows in the lesions over both forearm and palms which could not be appreciated by an unaided eye (Figure 1C,D). A circular scaling edge in the lesions termed as “Biett's sign” has been considered as a strong indicator of secondary syphilis.^4^ Although this feature was clinically evident on the lesions over palms in our patient (Figure 2B), dermoscopy was helpful to appreciate this feature in the smaller lesions (Figure 2C,D). We also noted that the scaling within the skin furrows starts to disappear from the center of the lesion as the lesions progress, ultimately forming a thin rim of scale in the periphery (Figure 2D). Orange background of lesions over palm seen in our patient is consistent with the description by Errichetti and Stinco,^3^ who attributed the finding to the deposition of hemosiderin in the dermis due to extravasation of erythrocytes. The secondary lesions of syphilis may be confused with the lesions of papulosquamous disorders like guttate psoriasis, pityriasis rosea, and pityriasis lichenoides chronica.^10^, ^11^, ^12^ Dermoscopy of guttate psoriasis usually displays dotted vessels distributed uniformly over the lesions while pityriasis lichenoides chronica commonly shows nondotted vessels, focally distributed dotted vessels, and orange‐yellowish structureless areas.^11^, ^12^ Pityriasis rosea shows peripheral whitish scaling as well as dotted vessels in an irregular or focal pattern with localized or diffuse yellowish‐orange structureless areas.^12^ Visualization of features like the color of the lesion, scaling, and vascular patterns using a dermoscope could help to differentiate between these conditions.

## 4 CONCLUSION

In atypical circumstances like ours, when the clinical suspicion of syphilis is low due to the absence of contact history and primary lesions, dermoscopy may provide important clues leading to serological tests and finally a proper diagnosis of secondary syphilis.

## CONFLICT OF INTEREST

None declared.

## AUTHOR CONTRIBUTION

MM and PA: collected clinical data and wrote the manuscript. AK and JS: contributed to patient evaluation and follow‐up. NK: reviewed the manuscript. All authors read and approved the final version of the manuscript.
